# Machine Learning and Optical-Coherence-Tomography-Derived Radiomics Analysis to Predict the Postoperative Anatomical Outcome of Full-Thickness Macular Hole

**DOI:** 10.3390/bioengineering11090949

**Published:** 2024-09-22

**Authors:** Yuqian Hu, Yongan Meng, Youling Liang, Yiwei Zhang, Biying Chen, Jianing Qiu, Zhishang Meng, Jing Luo

**Affiliations:** Department of Ophthalmology, The Second Xiangya Hospital of Central South University, Changsha 410011, China; huyuqian626@csu.edu.cn (Y.H.); mengyongan@csu.edu.cn (Y.M.); yony_liang@sina.cn (Y.L.); zyw5490515992022@163.com (Y.Z.); 8301180204@csu.edu.cn (B.C.); 8301180201@csu.edu.cn (J.Q.)

**Keywords:** macular hole, OCT-omics, machine learning, deep learning, personalized medicine

## Abstract

Full-thickness macular hole (FTMH) leads to central vision loss. It is essential to identify patients with FTMH at high risk of postoperative failure accurately to achieve anatomical closure. This study aimed to construct a predictive model for the anatomical outcome of FTMH after surgery. A retrospective study was performed, analyzing 200 eyes from 197 patients diagnosed with FTMH. Radiomics features were extracted from optical coherence tomography (OCT) images. Logistic regression, support vector machine (SVM), and backpropagation neural network (BPNN) classifiers were trained and evaluated. Decision curve analysis and survival analysis were performed to assess the clinical implications. Sensitivity, specificity, F1 score, and area under the receiver operating characteristic curve (AUC) were calculated to assess the model effectiveness. In the training set, the AUC values were 0.998, 0.988, and 0.995, respectively. In the test set, the AUC values were 0.941, 0.943, and 0.968, respectively. The OCT-omics scores were significantly higher in the “Open” group than in the “Closed” group and were positively correlated with the minimum diameter (MIN) and base diameter (BASE) of FTMH. Therefore, in this study, we developed a model with robust discriminative ability to predict the postoperative anatomical outcome of FTMH.

## 1. Introduction

Full-thickness macular hole (FTMH) is characterized by a complete disruption of the neurosensory retina from the inner limiting membrane (ILM) to the retinal pigment epithelium (RPE) in the center of the macula or fovea [1], leading to the impairment of central vision. The prevalence of FTMH varies from 0.2 to 0.8% [2,3,4], and it is more common in females and the elderly over 55 years old. Within five years of an FTMH occurrence in one eye, approximately 10–15% of contralateral eyes will develop an FTMH [5].

FTMH corresponds to stages 2–4 in the Gass classification, which categorizes the macular holes on the basis of biomicroscopic examination [6]. FTMH can also be categorized into three groups based on the size of the hole—small (<250 µm), medium (250–400 µm), and large (≥400 µm)—using measurements from optical coherence tomography (OCT) images [1]. Additionally, FTMH can be categorized according to its etiology. The primary FTMH, which is also called idiopathic FTMH, is initiated by vitreomacular traction (VMT), whereas the secondary FTMH is directly caused by trauma or associated disease (e.g., high myopia) without prior VMT [1]. 

Vitrectomy with ILM peeling and gas tamponade is regarded as the standard treatment of FTMH [7,8]. As reported, the anatomical closing rate of vitrectomy with ILM peeling surgery was over 80% [9,10,11], but it decreased to 56% for large FTMHs (≥400 µm) [12,13]. While the use of inverted ILM flaps in surgery has proven more effective than traditional ILM peeling for treating large and myopic FTMHs [9,14,15,16], the issue of FTMHs that fail to close still needs attention. Therefore, there is an urgent need to identify patients at high risk of postoperative failure to achieve anatomical closure and to develop personalized treatments accordingly.

Optical coherence tomography (OCT), which is commonly employed in clinical settings, can non-invasively offer retina images with high resolution. The OCT images facilitate precise visualization for FTMH diagnosis, measurement of macular holes, and prognosis analysis. Previous research has identified multiple prognostic markers for the surgical outcomes of macular holes, including the minimum diameter (MIN), base diameter (BASE), MH index (MHI), tractional hole index (THI), hole form factor (HFF), and diameter hole index (DHI) [12,17,18,19]. Nonetheless, the low repeatability of manual measurements of OCT parameters may remain a problem [20,21], which might limit their predictive ability. Consequently, an automated method for OCT macular hole measuring and analyzing is needed.

Radiomics is an innovative technology that can facilitate the extraction of high-throughput features from medical images [22]. It can contribute to clinical diagnosis and prognosis, as well as treatment efficacy assessment, offering significant potential in assisting clinical decision-making. “OCT-omics” is an approach combining “OCT” and “radiomics” and seeking to enhance the entire knowledge of retina in diseases, which has been raised by our team and used in predicting diabetic macular edema treatment response [23]. OCT-omics involves extracting and analyzing high-throughput quantitative features from OCT images, aiming to obtain a complete representation of retinal pathology.

The utilization of artificial intelligence (AI), such as via machine learning (ML) and deep learning (DL), in medical image analysis has great potential to improve the quality and reliability of predictive models [24,25]. ML models could help predict the anatomical outcome of FTMH patients, thus assisting retinal specialists in identifying those who are less likely to achieve anatomical closure. Consequently, this allows for the development of personalized treatments for individual patients.

To the best of the authors’ knowledge, there is currently no existing application of radiomics in predicting anatomical outcomes of FTMH. Previous research has primarily focused on idiopathic FTMH or certain types of secondary FTMH and used the manual measurements of OCT parameters, which were not comprehensive or representative enough to cover the practical clinical settings. 

In view of the limitations in current FTMH OCT parameters and anatomical outcome prediction, the objective of our study is to develop an ML model using radiomics features extracted from OCT images for the prediction of the postoperative anatomical outcomes of FTMH.

## 2. Materials and Methods

### 2.1. Study Population

This study was a retrospective study. Patients diagnosed with FTMH in the Second Xiangya Hospital of Central South University between January 2019 and August 2023 were eligible for inclusion.

The inclusion criteria were as follows: (1) confirmed diagnosis of FTMH using the spectral domain-OCT; (2) received vitreoretinal surgery for FTMH: vitrectomy and internal limiting membrane peeling, or vitrectomy with internal limiting membrane flap; (3) at least 1-month postoperative follow-up. The exclusion criteria were as follows: (1) existence of other ocular diseases causing vision loss such as severe cataract, glaucoma, and uveitis; (2) absence of follow-up OCT examination; (3) poor quality of OCT images.

Demographic data and preoperative clinical data included patient age, sex, best-corrected visual acuity (BCVA), and the existence of high myopia. And the preoperative BCVA was converted to LogMAR scores. Postoperative macular hole closure status was labeled based on the postoperative OCT images by two ophthalmologists and verified by a senior retina specialist. 

All eyes were divided randomly into a training set and a test set in a ratio of 7:3. 

### 2.2. OCT Image Acquisition

All eyes in this study underwent preoperative spectral-domain OCT (SD-OCT) scans using the Optovue RTVue XR Avanti system (Optovue Inc., Fremont, CA, USA). Radial scans (6 × 6 mm) centered on the fovea were performed, and among 18 radial B-scan images of each eye, the horizontal cross-sectional B-scan images were selected for this study. 

### 2.3. Region of Interest Segmentation and Parameter Measurement

All images were DICOM-formatted, and segmentation and feature extraction were conducted based on the formatted images. Region of interest (ROI) was manually selected from the inner limiting membrane (ILM) to the retinal pigment epithelium (RPE). ROI segmentation was performed using 3D Slicer software (version 5.0.3, https://www.slicer.org/, accessed on 6 January 2024) [26]. 

The preoperative OCT parameters of the macular holes including BASE, MIN, height of hole (H), and temporal and nasal arm length of hole (T and N) were manually measured using Fiji software (version 2.9.0, https://fiji.sc/, accessed on 15 May 2024). BASE was the diameter of FTMH at RPE level, while MIN was the minimum diameter of FTMH [12,17]. H was the distance between RPE and the innermost edge of the hole. T and N were the distances between the temporal endpoints or the nasal endpoints of MIN and BASE, respectively [18,19]. 

ROI segmentation and parameter measurement were performed independently by 2 ophthalmologists with 2 and 5 years of experience without being aware of patient groups or postoperative anatomical outcomes. Four indices were then calculated using the preoperative OCT parameters: HFF (T + N)/BASE), MHI (H/BASE), DHI (MIN/BASE), and THI (H/MIN). The ROI segmentation and parameter measurement results were reviewed and confirmed by a senior retina specialist with 30 years of experience.

### 2.4. Feature Extraction and Selection

In this study, we utilized a method called “OCT-omics”. Developed by our team [23], it involves feature extraction from OCT images using the radiomics technique, feature selection, and analysis. OCT-omics aims to give a complete representation of the retina in diseases with a large number of features.

Radiomics features were extracted from the segmented OCT images using the SlicerRadiomics extension in the 3D Slicer software [27]; OCT images were resampled to voxel dimensions of 1 × 1 × 1 mm^3^, and the intensity bin width value was 25.0. Eight types of the features were extracted: first-order statistics, shape features, gray-level co-occurrence matrix (GLCM), gray-level dependence matrix (GLDM), gray-level run length matrix (GLRLM), gray-level size zone matrix (GLSZM), neighborhood gray-tone difference matrix (NGTDM), and features based on wavelet transform. The radiomics features provided loads of information reflecting intensity, shape, texture, and spatial relationships in the OCT images. All features were standardized by z-score transformation in order to guarantee comparability and mitigate the impact of scale disparities. Forty eyes were randomly selected as a subgroup to calculate the intraclass correlation coefficient (ICC). Features with ICC values > 0.75 were considered to have good reproducibility. The Mann–Whitney U test (*p* < 0.05) was further performed to select features with significant distinctions between the “Closed” group and “Open” group. Subsequently, dummy variables were created for the existence of high myopia and surgery types. These selected OCT-omics features, along with the dummy variables representing the existence of high myopia and surgery types, were input to logistic regression models using a recursive feature elimination method to remove the features that contributed least to model performance.

### 2.5. Development of OCT-Omics Models 

Since the outcome (“Closed” and “Open”) distribution was imbalanced, the synthetic minority oversampling technique (SMOTE) [28] was carried out in the training set by utilizing the Scientific Platform Serving for Statistics Professional software (SPSSPRO, version 1.1.26, https://www.spsspro.com, accessed on 26 May 2024) to over-sample the minority class (Open) and balance the training set. The parameters of SMOTE were listed below:

- sampling_strategy = “auto”,
- random_state = None,
- k_neighbors = 5,
- n_jobs = None

After oversampling, a new training set was formed which contained 140 eyes from the original training set and 100 additional data from oversampling. The OCT-omics model was developed using the balanced new training set, and corresponding OCT-omics scores were calculated in every objective. The Mann–Whitney U test was employed in the balanced training set and test set to compare OCT-omics scores between the Close and Open groups. Pearson correlation analysis was conducted to examine the correlation between OCT-omics scores and preoperative OCT parameters of the macular hole. Decision curve analysis (DCA) was conducted to assess the clinical net benefits using the “dcurves” R package. Additionally, Kaplan–Meier analysis was conducted using the Sangerbox online tool (http://sangerbox.com/home.html, accessed on 7 July 2024) [29] to estimate the survival probability of the macular hole remaining open, with closure defined as the event of interest.

### 2.6. Training and Validation of Machine Learning Models

We trained a support vector machine (SVM) model and a back propagation neural network (BPNN) model using the selected OCT-omics feature of the new training set. The following were parameters of SVM and BPNN models:

- SVM: penalty factor C = 1; kernel function, linear; maximum of iterations = 1000; 5-fold cross-validation;
- BPNN: activation function, rectified linear unit (ReLU); solver, Limited-memory Broyden–Fletche–Goldfarb–Shanno (LBFGS); L2 regularization term = 1; number of iterations = 1000; 5-fold cross-validation.

The performance in classification and generalization capabilities of three classifiers—logistic regression, SVM, and BPNN—were evaluated. Performance metrics were calculated in the training and test sets, including accuracy, sensitivity (recall), specificity, positive predictive value (precision), negative predictive call, and the F1 score. Confusion matrices and ROC curves with corresponding area under the curve (AUC) values were visualized using the “ggplot2”, “reshape2”, and “pROC” R packages.

### 2.7. Statistical Analysis 

R software (version 4.3.2, https://www.R-project.org/, accessed on 30 May 2024) was used to perform data preprocessing and data visualization; Python (version 3.11.4, https://www.python.org/, accessed on 26 May 2024) was used to assist model development; and the Scientific Platform Serving for Statistics Professional software (SPSSPRO, version 1.1.26, https://www.spsspro.com, accessed on 26 May 2024) was used to perform model evaluation and statistical analysis. Continuous variables were presented as means ± standard deviations (SD) in normally distributed data or medians with interquartile ranges (IQR) in non-normally distributed data. Categorical variables were expressed as counts and percentages. To compare between groups, independent *t*-tests were performed in non-normally distributed data and Mann–Whitney U tests were performed in non-normally distributed data. To assess associations between categorical variables, Chi-square or Fisher’s exact tests were performed. The associations between continuous variables were evaluated using Pearson correlation analysis. All *p*-values were calculated from two-sided tests, with a significance threshold set at *p* < 0.05.

## 3. Results

### 3.1. Summary of Study Design and Baseline Characteristics

**Figure 1:**
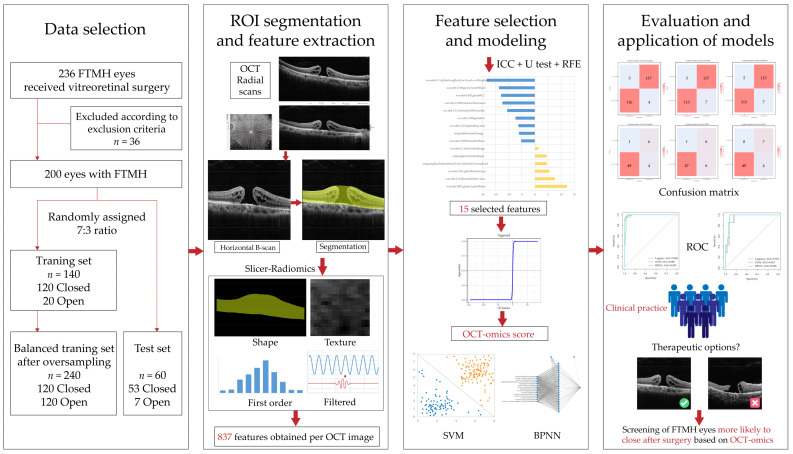
The workflow of the study.

The procedure of this study is depicted in Figure 1. It contained several key steps, including the data selection of the patient cohort, OCT image acquisition, ROI segmentation, feature extraction, feature selection, model development, and assessment [22].

This study included 200 eyes from 193 patients. Demographic and clinical characteristics of FTMH eyes are summarized in Table 1. The median age of the included patients was 61 years (IQR = 12). There were 133 (66.50%) eyes from female patients and 67 (33.50%) from male. There were no significant differences in baseline characteristics between the training and test sets.

### 3.2. OCT-Omics Model Development and Clinical Implication Evaluation

**Figure 2:**
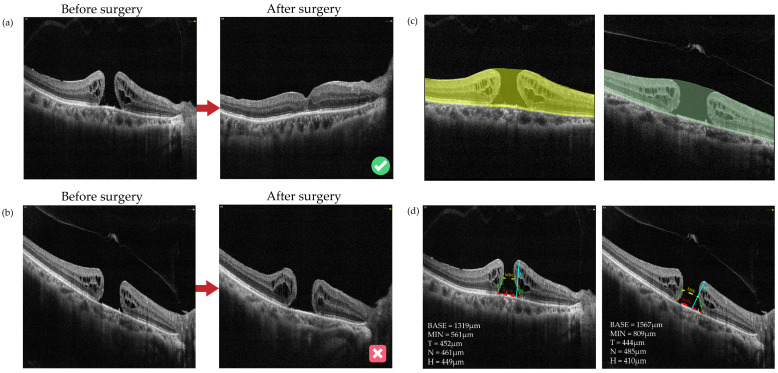
Images and ROI segmentation of typical cases in “Closed” and “Open” cases: (**a**) Typical cases of “Closed”: a 68-year-old male with FTMHs in the right eye who had received vitrectomy with ILM peeling surgery. The macular hole had been closed since the first follow-up which was the 12th day after surgery and kept closed to the latest follow-up (the 54th day after surgery). (**b**) Typical cases of “Open”: a 63-year-old female with FTMHs in the right eye who had received vitrectomy with ILM flap surgery. The macular hole had been staying open status at the latest follow-up, which was the 1080th day after surgery. (**c**) The ROI segmentation based on initial pretreatment OCT images for cases a and b. The ROI was selected from the inner limiting membrane (ILM) to the retinal pigment epithelium (RPE). (**d**) The preoperative OCT parameters of the macular hole for cases a and b, including BASE, MIN, height of hole (H), and temporal and nasal arm length of hole (T and N). The red dotted lines indicate BASE; the yellow dotted lines indicate MIN; the cyan lines indicate H; the green lines indicate T and N. BASE: base diameter of macular hole; MIN: minimal diameter of macular hole; N: nasal arm length of macular hole; T: temporal arm length of macular hole; H: height of macular hole.

There were 173 (86.50%) eyes labeled as “Closed” and 27 (13.50%) eyes labeled as “Open” after surgery. Figure 2 shows the typical cases of each group. The comparison of the baseline demographic and clinical characteristics of FTMH eyes between the two groups (“Closed” and “Open”) is presented in Table S1.

A total of 200 images in the dataset were divided into training and test sets. The training set contained 140 eyes, constituting 70% of the total dataset. In the training set, 53 images were labeled as “Closed” (88.33%) and 20 images were labeled as “Open” (14.29%). In the “Closed” group of the training set, the number of large, medium and small FTMHs were 67 (55.83%), 22 (18.33%), and 31 (25.83%), respectively. In the “Open” group of the training set, the number of large, medium and small FTMHs were 19 (95.00%), 1 (5.00%), and 0, respectively. The test set contained 60 eyes, constituting 30% of the total dataset. In the “Closed” group of the test set, the number of large, medium and small FTMHs were 32 (60.38%), 9 (16.98%), and 12 (22.64%), respectively. In the “Open” group of the test set, the number of large, medium and small FTMHs were 4 (57.14%), 2 (28.57%), and 1 (14.29%), respectively.

**Figure 3:**
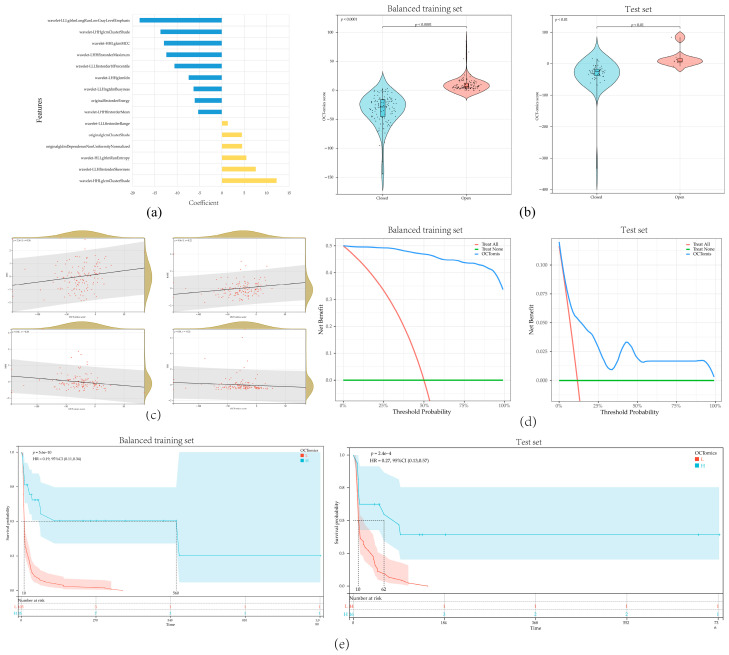
Evaluation and analysis of the OCT-omics model: (**a**) the distribution of coefficients for the 15 features included in the logistic regression model; (**b**) violin plots illustrating the comparison of OCT-omics scores between the “Closed” and “Open” groups in the training set and test set; (**c**) scatterplot presenting the correlation between OCT-omics score and BASE, MIN, MHI, and THI in the training set; (**d**) decision curve analysis curves for the OCT-omics model in the training set and test set; (**e**) Kaplan–Meier curves for the OCT-omics model in the training set and test set.

For each eye, 837 OCT-omics features were extracted. In the training set, a procedure of feature selection was conducted. First, 803 features were selected based on the criteria that ICC > 0.75. Then, after conducting the Mann–Whitney U test, 219 features were selected. Dummy variables were integrated to represent the presence of high myopia and surgery types. Following this, a new training set was created after using SMOTE for oversampling to balance the training set. The balanced new training set contained 140 eyes from the original training set and 100 additional data from oversampling. The recursive feature elimination method was used to create a logistic regression model (Table S2) with the combination of selected features and dummy variables. Finally, the model contained 15 key features, with the following equation (Figure 3a).

(1) $$\begin{array}{cc} OCTomics\;score & =-26.973\\ & +\;24.526\\ & \times \;originalgldmDependenceNonUniformityNormalized\\ & +\;7.552\times wavelet.LLHfirstorderSkewness\\ & -\;5.289\times wavelet.LHHfirstorderMean\\ & -\;13.716\times wavelet.LHHglcmClusterShade\\ & -\;7.408\times wavelet.LHHglcmIdn\\ & -\;10.585\times wavelet.LLLfirstorder10Percentile\\ & +\;1.307\times wavelet.LLLfirstorderRange\\ & -\;18.367\\ & \times \;wavelet.LLLglrlmLongRunLowGrayLevelEmphasis\\ & +\;4.464\times originalglcmClusterShade\\ & -\;6.333\times wavelet.LLHngtdmBusyness\\ & -\;12.931\times wavelet.HHLglcmMCC\\ & -\;12.405\times wavelet.LHHfirstorderMaximum\\ & -\;6.059\times originalfirstorderEnergy\\ & +\;12.2\times wavelet.HHLglcmClusterShade\\ & +\;5.455\times wavelet.HLLglrlmRunEntropy \end{array}$$

OCT-omics scores were significantly higher in the “Open” group compared to the “Closed” group in both the training set (*p* < 0.001) and the test set (*p* < 0.01) (Figure 3b). Additionally, OCT-omics scores exhibited positive correlations with BASE (*R* = 0.22, *p* < 0.001) and MIN (*R* = 0.26, *p* < 0.001) and presented negative correlations with MHI (*R* = −0.20, *p* = 0.02) and THI (*R* = −0.22, *p* = 0.01) (Figure 3c, Table A1). In both the training and test sets, DCA demonstrated that the OCT-omics model provides a higher clinical net benefit than the “Treat none” and “Treat all” strategy for most ranges of the threshold probabilities, suggesting that a meaningful clinical net benefit can be delivered by using OCT-omics score for prediction while reducing unnecessary surgical interventions (Figure 3d). In both sets, Kaplan–Meier curves showed that the MH postoperative open rates of the subgroup with high OCT-omics scores were higher than the subgroup with low OCT-omics scores at any time (Figure 3e), which suggested that FTMH with higher preoperative OCT-omics scores are less likely to close after surgery, suggesting that OCT-omics scores have great potential to predict the postoperative anatomical outcome in FTMH patients.

### 3.3. Evaluation of Overall Performance in Multiple Models

The three OCT-omics classification models, including logistic, SVM, and BPNN, exhibited strong discriminative capabilities in both the training and test sets. For the training set, the AUC of the logistic classifier was 0.998, with a sensitivity of 0.925, a specificity of 0.857, and an F1 score of 0.951. The AUC of the SVM classifier was 0.988, with a sensitivity of 0.942, a specificity of 0.975, and an F1 score of 0.958. The AUC of the BPNN classifier was 0.995, with a sensitivity of 0.942, a specificity of 0.958, and the F1 score was 0.950. For the test set, the classifiers also demonstrated good discriminative performance. The logistic classifier achieved an AUC of 0.941, and the sensitivity, specificity, and F1 scores were 0.925, 0.857, and 0.951, respectively. The SVM classifier reached an AUC of 0.943, with sensitivity, specificity, and F1 scores of 0.887, 0.857, and 0.931, respectively. The BPNN classifier achieved an AUC of 0.968, and the sensitivity, specificity, and F1 scores were 0.925, 1.000, and 0.961, respectively. The results are summarized in Table 2.

**Figure 4:**
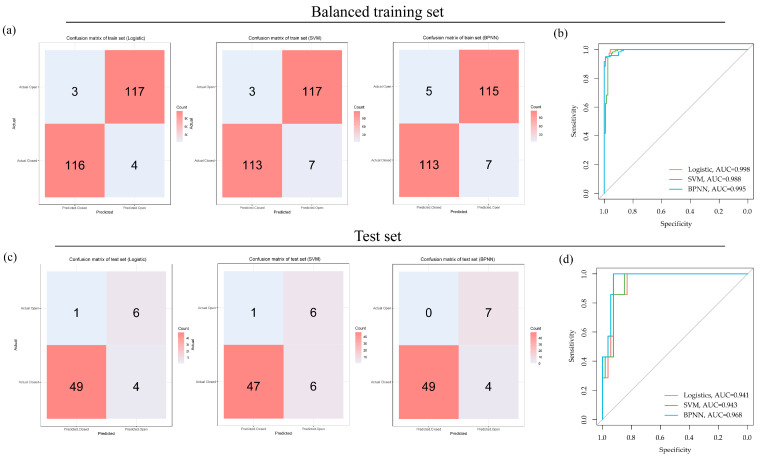
Classification performances of logistic, SVM, and BPNN models in the balanced training set and the test set: (**a**) confusion matrices for logistic, SVM, and BPNN models in the balanced training set; (**b**) ROC curves for logistic, SVM, and BPNN models in the balanced training set; (**c**) confusion matrices for logistic, SVM, and BPNN models in the test set; (**d**) ROC curves for logistic, SVM, and BPNN models in the test set.

High sensitivity values suggested efficacy in identifying positive cases (“Closed”), whereas high specificity values suggested efficacy in identifying negative cases (“Open”). F1 scores assess model performance on the basis of balancing specificity and sensitivity. The AUC values demonstrated the models’ ability to distinguish between “Closed” and “Open” subgroups based on OCT-omics features. Figure 4 showed the confusion matrices and ROC plots, visualizing the classification performances of the three models.

In conclusion, the OCT-omics classification models show great performance as well as potential clinical implications. These results demonstrate that OCT-omics can work as a non-invasive tool for predicting individualized anatomical outcomes of FTMH after surgery and assessing retinal pathologies, which has great potential for future research.

### 3.4. Clinical Implications of the OCT-Omics Model

**Figure 5:**
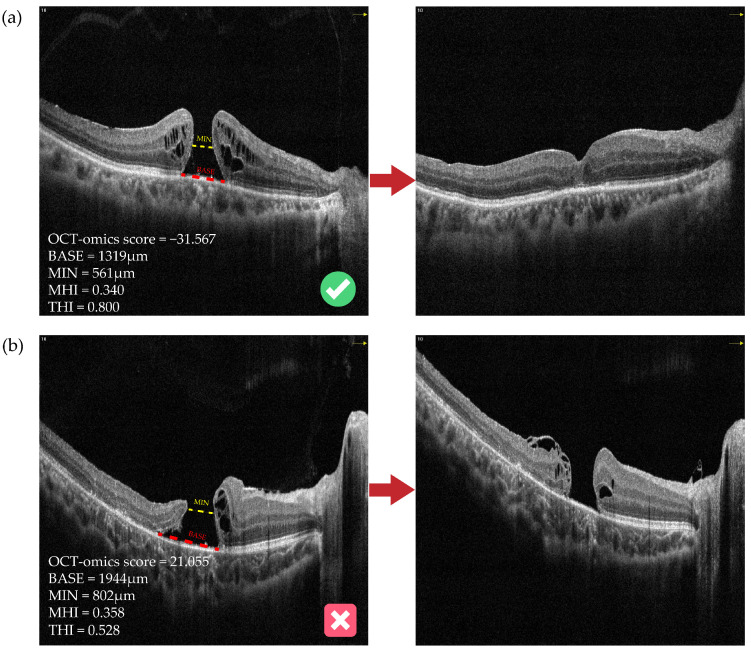
Preoperative and postoperative OCT images of two typical cases with FTMH. (**a**) An FTMH eye with an OCT-omics score of −31.567, which was classified as part of the “Closed” group by all of the logistic, SVM, and BPNN models, and the macular hole was observed to be closed at the first follow-up, which was the 12th day after surgery. (**b**) Another FTMH eye with an OCT-omics score of 21.055, which was classified as part of the “Open” group by all of the logistic, SVM, and BPNN models, and the macular hole was observed to stay open status at the latest follow-up, which was the 139th day after surgery. The red dotted lines indicate BASE; the yellow dotted lines indicate MIN. BASE: base diameter of macular hole; MIN: minimal diameter of macular hole; MHI: macular hole index; THI: tractional hole index.

Figure 5 shows preoperative and postoperative OCT images of two typical cases with FTMH involved in this study. The anatomical outcomes predictions based on the OCT-omics model correspond to the actual outcomes the actual outcomes. Figure 5a shows an FTMH eye of a 68-year-old male. The BASE of the macular hole was 1319 μm, and the MIN was 561 μm. The OCT-omics score calculated by the prediction model was −31.567, and this eye was classified as part of the “Closed” group with a rate of 0.999 predicted by the SVM model, and 0.998 by the BPNN model. Through the postoperative OCT images and patient records, we acknowledged that the macular hole of this patient had been closed since the first follow-up which was the 12th day after surgery (ILM peeling) and kept closed to the latest follow-up which was the 54th day after surgery. Figure 5b shows an FTMH eye of a 9-year-old boy. The BASE was measured as 1944 μm and MIN as 802 μm. The prediction model calculated the OCT-omics score to be 21.055, with a 0.006 probability for “Closed” predicted by the SVM model, and a 0.004 probability by the BPNN model. The latest follow-up of this patient, which was the 139th day after surgery (ILM flap), showed that the FTMH remained open.

## 4. Discussion

In this study, we employed machine learning and OCT-derived radiomics analysis to develop a model with which to predict the postoperative anatomical outcome of FTMH. We trained and evaluated the logistic, SVM, and BPNN models, with the results showing that all of the models performed well in the balanced training set and test set, suggesting the predictive capabilities of the preoperative OCT-omics model and highlighting the great potential of machine learning and OCT-derived radiomics analysis in predicting the anatomical outcome in FTMH patients after surgery. 

Unlike many previous studies that relied on a few OCT parameters, which are potentially insufficient for predicting postoperative outcomes [30], we employed radiomics to extract numerous features from OCT images and selected meaningful ones. This approach alleviates the issue of poor repeatability associated with manually measuring OCT parameters and accelerates the feature extraction process. We utilized the method of OCT-omics, which combines OCT images with radiomics analysis to enhance the knowledge of the retina in general. This approach has been developed by our team and utilized in predicting diabetic macular edema treatment response [23]. Based on OCT-omics, models were developed to predict the anatomical outcome of FTMH patients after surgery. The OCT-omics model can provide a quantitative and thorough assessment of the retina, presenting insights beyond mere macular hole parameters; thus, it may contribute additional information pertinent to the anatomical outcomes of FTMH. The results indicated that the OCT-omics classification model provides a promising example for personalized management strategies and holds the potential for assisting precision medicine.

The correlation between OCT-omics scores with BASE, MIN, MHI, and THI can somehow help with the interpretability of the OCT-omics model. Previous studies have found a clear negative relationship between BASE and MIN and post-operative anatomical outcome and visual acuity outcome of macular holes [31]. MHI and THI have been reported to be positively correlated with the vision outcome of FTMH [18,32]. Since the OCT-omics score is negatively correlative with MHI and THI, we can hypothesize that OCT-omics also makes sense in predicting the vision outcome of FTMH, but further research is needed to give strong evidence.

The choice of FTMH surgery types and the associated surgical techniques vary among surgeons when interventions are involved. Vitrectomy, together with ILM peeling and gas tamponade, is regarded as the standard process in treating FTMH. Reports indicate that the anatomical closure rate after vitrectomy with ILM peeling surgery exceeds 80% [9,10,11]; however, this rate declines to 56% for large FTMHs (≥400 µm) [12,13]. The adoption of inverted ILM flaps in surgery has demonstrated higher closing rates compared to ILM peeling for treating large and myopic FTMHs [9,14,15,16]. In our study, we observed that BASE varied among different surgical types (*p* = 0.035) (Table A2), indicating that surgeons consider macular hole size when deciding the surgical type. And in our study, the surgeries were conducted by the same retina specialist, thereby minimizing the bias caused by surgical techniques. 

We have listed the predictive performance of our three developed models and other state-of-the-art models in Table 3. All of the data represent the predictive performance of test sets, respectively. Xiao et al. (2021) [33] developed the models to predict the postoperative anatomical outcome of full-thickness idiopathic MH (IMH), and the random forest algorithm performed best with the AUC of 0.951 in the test set. Zgolli et al. (2022) [34] constructed a model using the medical decision support system (MDSS) algorithm with an AUC of 0.967 in the test set. Xiao et al. (2023) [35] developed multimodal deep fusion network (MDFN) models to classify the etiology and predict the anatomical outcomes of full-thickness idiopathic IMH or FTMH, resulting in an AUC of 0.947 in predicting the outcome of full-thickness IMH and 0.904 in FTMH. The AUC of the BPNN model we have developed was the highest and indicated good predictive capacity of the model. However, the lack of a validation set of our models remains a defect. Therefore, future research with larger datasets is still needed.

This study is subject to several limitations. First, as a retrospective study conducted at a single center, there may exist possible biases such as selection bias, incomplete data, and confounding variables affecting the generalizability of the results to broader populations or other healthcare settings. Second, the evaluation of the predictive capacities in three models was not performed separately for the three groups in different FTMH sizes (small, medium, large FTMHs) because the numbers of patients with medium and small FTMHs in our dataset (Table 1), especially in the test set, were small and not suitable for separate analysis. Although the radiomics features we selected contained information on FTMH size, and the OCT-omics score was proved to be positively correlative with MIN of FTMH, which was the basis for the classification of small, medium and large FTMHs, future research with larger datasets is still needed for more specific analysis and the separate evaluation of groups with different FTMH sizes. Third, the NPVs for the test set in each model were low, mainly because of high closure rates after surgery of FTMHs (80% for all FTMHs and 56% for large FTMHs, as reported). The “Open” group accounts for only 11.67% (7 eyes) in the test set so the NPVs for the test set were not easy to increase. Fourth, there are clinical features which are not included, such as duration and the practice of postoperative prone posturing. The duration is a known predictor of FTMH outcomes, but it was not consistently documented in our patient records. Meanwhile, reports suggest that duration mainly influences visual outcomes [36]; hence, duration data were omitted from our analysis. The postoperative prone posturing refers to maintaining a face-down position after surgery for two weeks, which has been reported to be beneficial for the closing of large FTMH, but no significant advantages have been observed in small or medium FTMH [37]. However, for a similar reason, it is hard to identify patients’ positions in this retrospective study. Fifth, this study exclusively addressed anatomical outcomes, neglecting visual outcomes. As the predictors for visual outcomes may differ from those of anatomical outcomes, they were not analyzed concurrently.

In subsequent research, it is imperative to conduct prospective studies with larger patient cohorts to validate and enhance our predictive model. Comprehensive medical record information and follow-up data are needed for extensive research. Additionally, to augment the generalizability of the model, recruiting patients from multiple centers is crucial. A more detailed description of macular hole and retina lesions, such as the perifoveal pseudocyst area [38], can be taken into account to increase the interpretability of the model. Furthermore, with expanded patient cohorts, the different subtypes of FTMH, particularly myopic FTMH, could be analyzed separately, and the predictive ability of OCT-omics models on vision outcome of FTMH is to be analyzed in the future.

## 5. Conclusions

In conclusion, this study employed the “OCT-omics” approach, integrating radiomics analysis of OCT images to assess the retina comprehensively and predict the anatomical outcome of FTMH. The established radiomics model holds promising predictive abilities to predict FTMH anatomical outcomes after surgery, thus assisting clinical decision-making and precision medicine. For future research, larger datasets and prospective clinical trials will be especially beneficial for the model construction.

## Figures and Tables

**Table 1:** Baseline demographic and clinical characteristics of FTMH eyes in training and test sets.

Variables	Total (*n* = 200)	Training Set (*n* = 140)	Test Set (*n* = 60)	*p*
Age, years, M (IQR)	61.00 (12.00)	61.00 (13.25)	61.00 (10.00)	0.393 ^f^
Age groups, n (%)				0.086 ^g^
<40	17 (8.50)	2 (2.33)	15 (10.71)	
≥40	183 (91.50)	58 (96.67)	125 (89.29)	
Sex, n (%)				0.492 ^g^
Female	133 (66.50)	91 (65.00)	42 (70.00)	
Male	67 (33.50)	49 (35.00)	18 (30.00)	
Laterality, n (%)				0.951 ^g^
Right	106 (53.00)	74 (52.86)	32 (53.33)	
Left	94 (47.00)	66 (47.14)	28 (46.67)	
Outcome, n (%)				0.619 ^g^
Closed	173 (86.50)	53 (88.33)	120 (85.71)	
Open	27 (13.50)	20 (14.29)	7 (11.67)	
High myopia, n (%)				0.387 ^g^
No	170 (85.00)	121 (86.43)	49 (81.67)	
Yes	30 (15.00)	19 (13.57)	11 (18.33)	
Surgery, n (%)				0.055 ^g^
ILM peeling ^a^	136 (68.00)	101 (72.14)	35 (58.33)	
ILM flap ^b^	64 (32.00)	39 (27.86)	25 (41.67)	
Size of FTMH ^c^, n (%)				0.947 ^g^
Large	122 (61.00)	36 (60.00)	86 (61.43)	
Medium	43 (17.00)	11 (18.33)	23 (16.43)	
Small	44 (22.00)	13 (21.67)	31 (2214)	
Examination interval ^d^, day, M (IQR)	59.00 (194.25)	70.50 (230.75)	55.50 (168.5)	0.156 ^f^
BCVA (logMAR) ^e^, M (IQR)	1.00 (0.57)	1.00 (0.60)	0.92 (0.40)	0.895 ^f^
BASE, μm, M (IQR)	965.50 (591.00)	953.00 (546.5)	1059.50 (598.5)	0.570 ^f^
MIN, μm, M (IQR)	478.00 (377.75)	478.00 (318.50)	486.00 427.75)	0.657 ^f^
N, μm, M (IQR)	330.50 (198.00)	334.00 (195.25)	327.00 (215.75)	0.915 ^f^
T, μm, M (IQR)	325.00 (196.75)	327.00 (195.25)	318.00 (214.25)	0.832 ^f^
H, μm, M (IQR)	428.50 (108.50)	428.00 (106.00)	430.50 (126.25)	0.719 ^f^
HFF, M (IQR)	0.73 (0.27)	0.72 (0.26)	0.74 (0.27)	0.897 ^f^
MHI, M (IQR)	0.44 (0.24)	0.45 (0.23)	0.43 (0.24)	0.617 ^f^
DHI, M (IQR)	0.51 (0.27)	0.51 (0.26)	0.47 (0.28)	0.542 ^f^
THI, M (IQR)	0.92 (0.90)	0.88 (0.86)	0.95 (0.96)	0.996 ^f^

^a^ vitrectomy with internal limiting membrane peeling and gas tamponade; ^b^ vitrectomy with internal limiting membrane flap and gas tamponade; ^c^ FTMHs were divided into three groups based on the MIN: small (<250 µm), medium (250–400 µm), and large (≥400 µm); ^d^ intervals between the preoperative OCT examination and the latest OCT examination assessing the effect of the surgery; ^e^ preoperative BCVA was converted to LogMAR scores; ^f^ calculated using Mann–Whitney U test; ^g^ calculated using Chi-square test. ILM: internal limiting membrane; BCVA: best-corrected visual acuity; BASE: base diameter of macular hole; MIN: minimal diameter of macular hole; N: nasal arm length of macular hole; T: temporal arm length of macular hole; H: height of macular hole; HFF: hole form factor; MHI: macular hole index; DHI: diameter hole index; THI: tractional hole index; n: number; μm: micrometer; M: Median; IQR: interquartile range.

**Table 2:** Performances of models to predict anatomical outcomes of FTMH.

	Training Set			Test Set		
	Logistic	SVM	BPNN	Logistic	SVM	BPNN
SEN	0.967	0.942	0.942	0.925	0.887	0.925
SPE	0.975	0.975	0.958	0.857	0.857	1.000
ACC	0.971	0.958	0.950	0.917	0.883	0.933
PPV	0.975	0.974	0.958	0.980	0.979	1.000
NPV	0.967	0.944	0.943	0.600	0.500	0.636
F1	0.971	0.958	0.950	0.951	0.931	0.961
AUC	0.998	0.988	0.995	0.941	0.943	0.968

SEN: sensitivity; SPE: specificity; ACC: accuracy; PPV: positive predictive value; NPV: negative predictive value; F1: F1 score; AUC: area under the receiver operating curve.

**Table 3:** Predictive performance in the state-of-the-art machine learning models.

	Algorithm	MH	Surgery Type	Eyes (Patients) (n)	Country	SEN	SPE	ACC	AUC
Xiao (2021) [33]	Random forest	Full-thickness IMH	ILM peeling	288	China	0.973	0.904	0.892	0.951
ZGOLLI (2022) [34]	MDSS	Full-thickness IMH	IML flap	120 (114)	Tunis	\	\	\	0.967
Xiao (2023) [35]	MDFN	Full-thickness IMH	ILM peeling	209	China	0.979	0.815	0.875	0.947
Xiao (2023) [35]	MDFN	FTMH	ILM peeling	330	China	0.766	0.977	0.825	0.904
Our models	Logistic	FTMH	ILM peeling or IML flap	200 (193)	China	0.925	0.857	0.917	0.941
	SVM					0.887	0.857	0.883	0.943
	BPNN					0.925	1	0.933	0.968

MDSS: medical decision support system; MDFN: multimodal deep fusion network; IMH: idiopathic MH; FTMH: full-thickness macular hole; ILM: internal limiting membrane; SEN: sensitivity; SPE: specificity; ACC: accuracy; AUC: area under the receiver operating curve.

## Data Availability

The source data and R scripts used for analysis in this study are available upon reasonable request to the corresponding author.

## Supplementary Materials

The following supporting information can be downloaded at https://www.mdpi.com/article/10.3390/bioengineering11090949/s1: Table S1: Baseline demographic and clinical characteristics of FTMH eyes in “Closed” and “Open” groups; Table S2: Key features selected for logistic regression model.

## Appendix A

**Table A1:** Correlation coefficient of Spearman correlation analysis between OCT-omics score and OCT parameters in the training set.

	BASE	MIN	N	T	H	HFF	MHI	DHI	THI	OCT-Omics
BASE	1 ***	0.653 ***	0.719 ***	0.766 ***	0.324 ***	−0.131	−0.805 ***	−0.2 **	−0.442 ***	0.217 ***
MIN	0.653 ***	1 ***	0.141 *	0.22 ***	−0.04	−0.658 ***	−0.718 ***	0.518 ***	−0.904 ***	0.255 ***
N	0.719 ***	0.141 *	1 ***	0.865 ***	0.52 ***	0.46 ***	−0.411 ***	−0.591 ***	0.074	0.153 *
T	0.766 ***	0.22 ***	0.865 ***	1 ***	0.557 ***	0.398 ***	−0.437 ***	−0.532 ***	0.016	0.137
H	0.324 ***	−0.04	0.52 ***	0.557 ***	1 ***	0.442 ***	0.208 **	−0.384 ***	0.39 ***	0.103
HFF	−0.131	−0.658 ***	0.46 ***	0.398 ***	0.442 ***	1 ***	0.436 ***	−0.733 ***	0.787 ***	−0.103
MHI	−0.805 ***	−0.718 ***	−0.411 ***	−0.437 ***	0.208 **	0.436 ***	1 ***	−0.067	0.737 ***	−0.2 **
DHI	−0.2 **	0.518 ***	−0.591 ***	−0.532 ***	−0.384 ***	−0.733 ***	−0.067	1 ***	−0.646 ***	0.055
THI	−0.442 ***	−0.904 ***	0.074	0.016	0.39 ***	0.787 ***	0.737 ***	−0.646 ***	1 ***	−0.215 **
OCT-omics	0.217 ***	0.255 ***	0.153 *	0.137	0.103	−0.103	−0.2 **	0.055	−0.215 **	1 ***

BASE: base diameter of macular hole; MIN: minimal diameter of macular hole; N: nasal arm length of macular hole; T: temporal arm length of macular hole; H: height of macular hole; HFF: hole form factor; MHI: macular hole index; DHI: diameter hole index; THI: tractional hole index; ***, **, and * represent *p* < 0.001, *p* < 0.05, and *p* < 0.1, respectively.

**Table A2:** BASE of FTMHs with different surgical types.

Variables	Total (n = 200)	ILM Peeling (n = 136)	ILM Flap (n = 64)	Statistic	*p*
BASE, M (IQR)	965.50 (591)	914.00 (565)	1029.00 (574)	Z = −2.10	0.035 **

BASE: base diameter of macular hole; ILM: internal limiting membrane; n: number; Z: Mann–Whitney test; M: Median; IQR: interquartile range; ** represents *p* < 0.05
