# Supplementary material for: Machine Learning and Optical-Coherence-Tomography-Derived Radiomics Analysis to Predict the Postoperative Anatomical Outcome of Full-Thickness Macular Hole
Source: Bioengineering (Basel). 2024 Sep 22;11(9):949. doi: 10.3390/bioengineering11090949 (PMC11428902; doi:10.3390/bioengineering11090949)
Supplement: Supplementary file 1 [file bioengineering-11-00949-s001.zip › Table S1-Baseline demographic and clinical characteristics of FTMH eyes in “Closed” and “Open” groups.pdf]

Table S1. Baseline demographic and clinical characteristics of FTMH eyes in "Closed" and "Open" groups.

Variables	Total (n = 200)	"Closed" (n = 173)	"Open" (n = 27)	*p*
Age, years, M (IQR)	61.00 (12.00)	61.00 (12.00)	60.00 (10.00)	0.926 [f]
Age group, n (%)				1.000 [g]
< 40	17 (8.50)	15 (8.67)	2 (7.41)	
≥ 40	183 (91.50)	158 (91.33)	25 (92.59)	
Sex, n (%)				0.195 [g]
Female	133 (66.50)	118 (68.21)	15 (55.56)	
Male	67 (33.50)	55 (31.79)	12 (44.44)	
Laterality, n (%)				0.898 [g]
Right	106 (53.00)	92 (53.18)	14 (51.85)	
Left	94 (47.00)	81 (46.82)	13 (48.15)	
High myopia, n (%)				**0.046** [g]
No	170 (85.00)	151 (87.28)	19 (70.37)	
Yes	30 (15.00)	22 (12.72)	8 (29.63)	
Surgery type, n (%)				0.242 [g]
ILM peeling [a]	136 (68.00)	115 (66.47)	21 (77.78)	
ILM flap [b]	64 (32.00)	58 (33.53)	6 (22.22)	
Size of FTMH [c], n (%)				**0.015** [g]
Large	122 (61.00)	99 (57.23)	23 (85.19)	
Medium	43 (17.00)	31 (17.92)	3 (11.11)	
Small	44 (22.00)	43 (24.86)	1 (3.70)	
Examination interval [d], day, M (IQR)	59.00 (194.25)	56.00 (154)	62.00 (252)	0.223 [f]
BCVA (logMAR) [e], M (IQR)	1.00 (0.57)	0.92 (0.52)	1.22 (0.53)	**0.027** [f]
BASE, μm, M (IQR)	965.50 (591.00)	921.00 (532)	1251.00 (613)	**<0.001** [f]
MIN, μm, M (IQR)	478.00 (377.75)	449.00 (345)	641.00 (294.5)	**<0.001** [f]
N, μm, M (IQR)	330.50 (198.00)	325.00 (203)	444.00 (239)	**0.012** [f]
T, μm, M (IQR)	325.00 (196.75)	312.00 (207)	402.00 (205)	**0.023** [f]
H, μm, M (IQR)	428.50 (108.50)	430.00 (110)	426.00 (92.5)	0.750 [f]
HFF, M (IQR)	0.73 (0.27)	0.74 (0.27)	0.65 (0.21)	0.056 [f]
MHI, M (IQR)	0.44 (0.24)	0.46 (0.23)	0.36 (0.13)	**<0.001** [f]
DHI, M (IQR)	0.51 (0.27)	0.50 (0.27)	0.52 (0.16)	0.969 [f]
THI, M (IQR)	0.92 (0.90)	0.98 (0.94)	0.64 (0.34)	**0.003** [f]

[a] Vitrectomy with internal limiting membrane peeling and gas tamponade;

[b] Vitrectomy with internal limiting membrane flap and gas tamponade;

[c] FTMHs were divided into three groups based on the MIN: small (< 250 μm), medium (250-400 μm), and large (⩾ 400 μm)

[d] Intervals between the preoperative OCT examination and the latest OCT examination assessing the effect of the surgery

[e] Preoperative BCVA was converted to LogMAR scores;

[f] Calculated using Mann-Whitney U test;

[g] Calculated using Chi-square test;

ILM: internal limiting membrane; BCVA: best-corrected visual acuity; BASE: base diameter of macular hole; MIN: minimal diameter of macular hole; N: nasal arm length of macular hole; T: temporal arm length of macular hole; H: height of macular hole; HFF: hole form factor; MHI: macular hole index; DHI: diameter hole index; THI: tractional hole index; n: number; μm: micrometer; M: Median; IQR, interquartile range.
